# Bioactive components and clinical potential of *Astragalus* species

**DOI:** 10.3389/fphar.2025.1585697

**Published:** 2025-05-16

**Authors:** Shuo Li, Xunpeng Hu, Fen Liu, Weiming Hu

**Affiliations:** ^1^ Gansu Phamaceutical Industry Innovation Research Institute, Key Laboratory of Quality and Standard of TCM of Gansu Province, Gansu University of Chinese Medicine, Lanzhou, China; ^2^ College of pharmacy, Gansu University of Chinese Medicine, Lanzhou, China; ^3^ Jiangxi Provincial Key Laboratory of Plant Germplasm Resources Innovation and Genetic Improvement, Lushan Botanical Garden, Jiangxi Province and Chinese Academy of Sciences, Jiujiang, China; ^4^ Jiangxi Academy of Forestry, Nanchang, China

**Keywords:** bioactive molecules, *Astragalus*, pharmacological action, mechanism of action, medicine and food homology (MFH)

## Abstract

*Astragalus* L., the world’s largest vascular plant genus, has been used medicinally and as food for centuries, especially in traditional Chinese medicine It is widely applied in immune modulation, antioxidant therapy, anti-inflammatory treatments, and antitumor applications. Recent studies show that *Astragalus* species is rich in bioactive compounds, such as polysaccharides, flavonoids, saponins, alkaloids, and simple phenolics, which demonstrate significant pharmacological effects, including anti-inflammatory, antioxidant, immunomodulatory, and antitumor properties, along with potential benefits for Alzheimer’s disease and diabetes. This review synthesizes 140 references to analyze 51 newly identified flavonoids, 31 triterpenoid saponins, and 19 alkaloids in *Astragalus* (2020–2025), focusing on their chemical structures and bioactivities. It also examines *Astragalus* species in medicine and food homology (MFH) and how processing methods affect its efficacy. Furthermore, the mechanisms behind its anti-inflammatory, antioxidant, immune-boosting, antitumor, neuroprotective, and hypoglycemic effects are discussed. Future studies should prioritize large-scale clinical trials to confirm *Astragalus*’s efficacy and safety, explore combination therapies, and improve sustainable resource utilization to expand its medical and food applications. Keywords: Bioactive molecules, *Astragalus*, Pharmacological action, Mechanism of action, Medicine and food homology (MFH).

## 1 Introduction

*Astragalus* is a genus within the legume family and is recognized as the largest vascular genus in the world, comprising approximately 2,900 species (Amiri et al., 2020). There are two primary locations in which *Astragalus* species: are distributed the Asia–Europe region and the Americas. Nearly 80% of species are found in the Asia–Europe region, amounting to approximately 2,400 species, and more than 500 species are confined to the Americas (Amiri et al., 2020). *Astragalus* species have a diverse range of applications, including medicinal uses; in food, animal feed, and fuel; and for ornamental purposes, particularly due to their significant nutritional and therapeutic value (Liu et al., 2017). China has a long history of utilizing *Astragalus*, which was first documented in the *Shennong Materia Medica*. Evidence suggests that the use of *Astragalus* dates back to the Qin and Han dynasties (221 BC - AD 202). Currently, there are 278 recognized species of *Astragalus* in China, along with two subtypes and 35 variants (Institute of Botany, 2009-2025). These plants are found in various provinces and regions in both northern and southern China, particularly in Tibet (Himalayan Mountains), Central Asia, and Northeast China (Institute of Botany, 2009–2025). According to the *China Pharmacopoeia 2020 Edition*, only *Astragali Radix* (AR, Huangqi in Chinese, dried root of *Astragalus membranaceus* var. *mongholicus* (Bge) Hsiao or *Astragalus membranaceus* (Fisch.) Bge.) and *Astragali Complanati Semen* (ASC) (Dried ripe seed of *Astragalus complanatus* R. Br) are permitted for use as nationally recognized medicinal drugs (Committee, 2020). However, throughout history, many other plants have been used in place of *Astragalus membranaceus*, such as *Astragalus tongolensis* Ulbr. (Wu and He, 1992), *Astragalus floridulus* Podlech, *Astragalus chrysopterus* Bunge, *Astragalus hoantchy* Franch., *Astragalus purpurinus* (Y. C. Ho) Podlech and L. R. Xu, and *Hedysarum polybotrys* Hand.-Mazz.

To date, over 300 chemical constituents have been isolated from *Astragalus*, including polysaccharides, Lignin (Obaid et al., 2023), flavonoids, alkaloids, coumarin (Mohamed et al., 2007) saponins, steroids, organic acids, volatile oils (Gad et al., 2021), and other bioactive compounds (Li et al., 2014). *Astragalus* species contain a variety of biologically active substances and nutrients, including polysaccharides and flavonoids, and other tonic components. *Astragalus* and its medicinal components exhibit a wide range of pharmacological effects, including immunomodulation, regulation of gastrointestinal motility, gastrointestinal protection, enhancement of cardiac function, promotion of hematopoietic function, neuroprotection, and protection of the lungs, liver, and kidneys (BiChen et al., 2022; Fengyan et al., 2022; Qianbo et al., 2022; Xinyi et al., 2022; Kagemasa et al., 2023; Yi et al., 2023). Additionally, the bioactive components of *Astragalus* possess antioxidant properties, anti-inflammatory effects, anti-fatigue capabilities, hypoxia resistance, and the ability to lower blood sugar and blood lipids, in addition to anti-tumor and antiviral activities (Wenfang et al., 2020; Xintong et al., 2023). These attributes suggest significant their therapeutic potential for conditions such as peptic ulcers, heart failure, diabetes, Alzheimer’s disease, chronic obstructive pulmonary disease (COPD), and obesity (Ying-jie et al., 2020; Maria et al., 2023; Chongyang et al., 2024). Recognized as a valuable traditional Chinese medicine, *Astragalus* is noted for its mild flavor and role in strengthening the spleen and stomach while tonifying both Qi (The intangible, high-mobility nutritive substance that maintains vital activities.) (IHS and Traditional Chinese Medicine, 2025) and blood. Since 2002, it has been included in the list of homologous foods and drugs issued by the Ministry of Health of China. They consist of nearly 90% protein (polypeptides), including seven essential amino acids such as lysine and leucine (Ran, 2012). Furthermore, they contains calcium, iron, and 14 trace elements, including magnesium, zinc, copper, and manganese, as well as vitamins A1, B1, B2, and B3. These properties contribute to its reputation as both a food and medicinal resource often utilized in tea, wine, soup, porridge, pastes, vegetables, and hot pot dishes, and dietary supplements (Butkutė et al., 2017; Anim Okyere et al., 2021).

Although *Astragalus* L. have demonstrated extensive pharmacological activities in both traditional medicine and modern research, the mechanisms of action of their specific bioactive components, the chemical variations among different species, and their relationship with therapeutic efficacy remain incompletely elucidated. Furthermore, critical gaps persist in current research, including the safety of *Astragalus* species in MFH applications, the impact of optimal processing methods on active constituents, and the lack of large-scale clinical evidence. Therefore, this review systematically summarizes the chemical composition of *Astragalus* species in recent years (2020–2025), highlights current research hotspots regarding their pharmacological effects and mechanisms, and explores their potential in chronic disease treatment and functional food development. The aim is to provide a theoretical foundation and practical guidance for future research.

## 2 Methodology

A comprehensive literature search was conducted in scientific databases including PubMed, Web of Science, ScienceDirect, Google Scholar, WanfangData, Chinese Pharmacopoeia, and China National Knowledge Infrastructure (CNKI) using keywords such as *Astragalus*, *Astragalus* L., *Astragalus* spp., *Astragalus*, *Astragali radix*, and *Astragali complanati* semend. Chemical structures of identified metabolites were drawn using ChemDraw software. This study systematically integrated the phytochemical composition, biological activities, pharmacological effects, dual use in medicine and food applications, and clinical research of the *Astragalus* genus. The literature review spanned an unrestricted timeframe, with chemical component-related literature primarily published from 2020 to 2025.

## 3 Structural characteristics and physicochemical properties of *Astragalus* species

### 3.1 Flavonoids

Flavonoids are among the primary components of *Astragalus* species. Flavonoids exist in various forms in *Astragalus* species. Some are present in their free form, while others are conjugated with sugars to form glycoside compounds. For instance, calycosin can exist independently, and there are corresponding glycoside compounds present within the plant tissue (Li W. et al., 2019).

**FIGURE 1:**
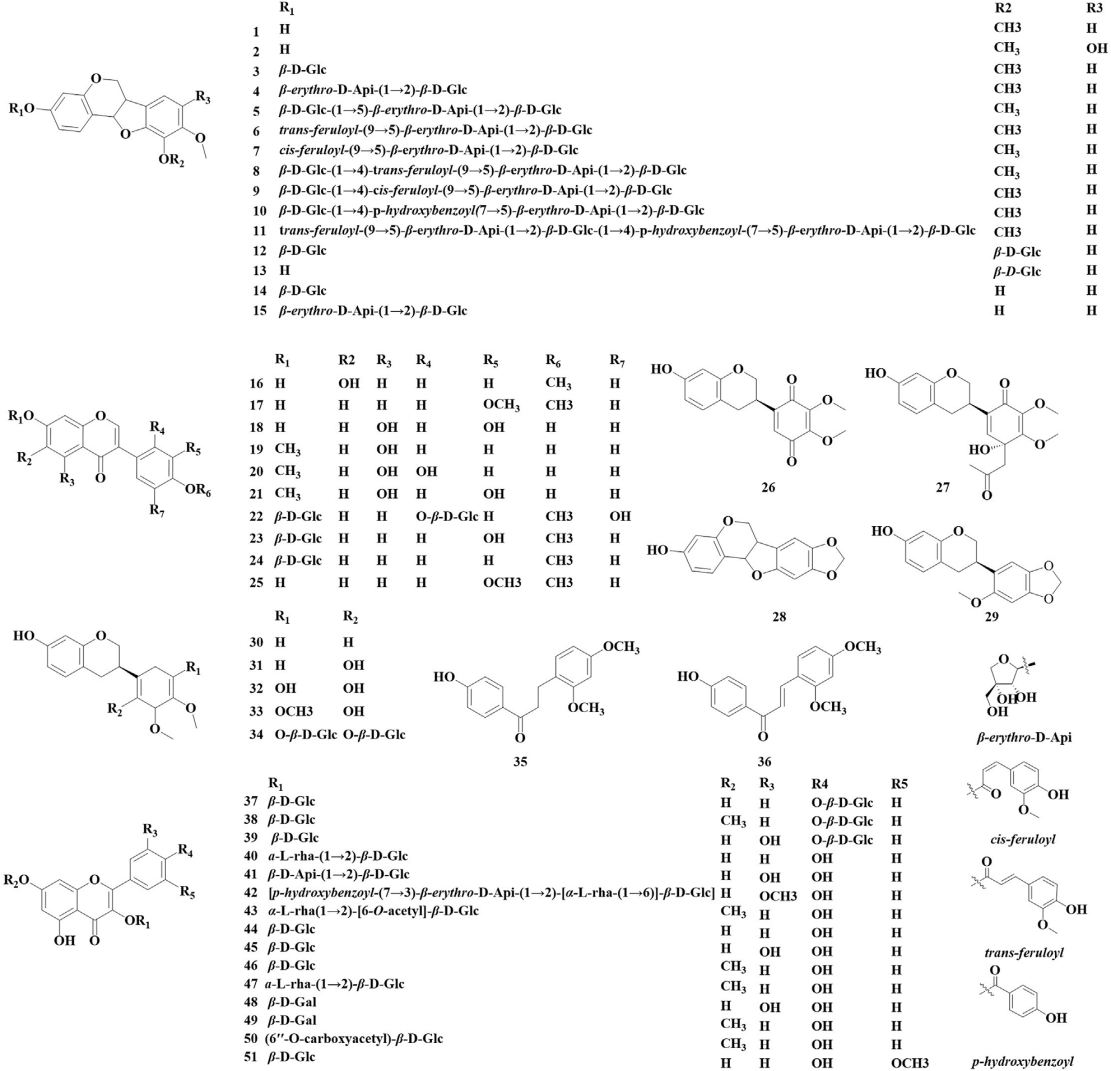
Flavonoid compounds identified in *Astragalus* species over the past 5 years.

Flavonoid compounds, as significant components of the chemical composition of *Astragalus*, encompass a variety of types, including flavonoids, flavonols, isoflavones, dihydroflavonoids, and dihydroflavones (Li et al., 2019). Over the past 5 years, a total of 51 flavonoids have been identified in the *Astragalus* species, including 26 isoflavones (1–2, 3–15, 16, 17–24, 25, 28), 15 flavonols (37–51), isoflavans (26, 27, 29–34), and two chalcone (35–36) (Figure 1; Table 1). These compounds exhibit various biological activities, including anti-cancer properties and antioxidant effects; inhibit tyrosine activity; modulate *α*-glucosidase activity; and suppress high-sugar-induced inflammatory responses in HK-2 cells. Şahin Yağlioğlu et al. (2022) obtained a spill-deoffuarian from the aboveground part of *Astragalus leucothrix*. This compound was evaluated for its anti-cancer and cytotoxic activity against HeLa cells and Rat glioma cells (C6 cells). The results indicated that the compound exhibited strong anti-cancer activity, with an IC_50_ value of 2.81 ± 0.00 μM, and their cytotoxicity was comparable to that of 5-FU. Manh Khoa et al. (2023) isolated a novel isoflavane derivative (27) from the aerial parts of *A. membranaceus*. In comparison to a positive control, kojic acid, compound 30 demonstrated a significant inhibitory effect on tyrosinase, with an IC_50_ value of 42.4 ± 1.3 μM at a concentration of 100 μM*.* Bao et al. (2022) isolated 11 new isoflavone derivatives (4–13, 15) and three known isoflavone derivatives (1, 3, 14) from the roots of *A. membranaceus* var. *mongholicus.* They utilized *α*-glucosidase to screen these compounds for their ability to reduce blood sugar, which revealed that compounds one and six partially inhibit *α*-glucosidase. Additionally, the method of evaluating antioxidant activity was refined, demonstrating that at a concentration of 12.5 μM, the oxidative free radical absorption capacity of compounds 1, 6, 7, 11, and 13–15 surpassed that of Trolox. Furthermore, compounds 6 and 14 were found to demonstrate DPPH free radical scavenging activity, with IC_50_ values ranging from 43.9 to 51.7 μM. Khalfallah et al. (2023) conducted a study on the ground portion of *Astragalus armatus* subsp. *numidicus* (Murb.) and identified a new glycosyls in (42). Xiao (Xiao, 2024) isolated a new isoflavone compound (22), a new isoflavone derivative (12), two isoflavones (23, 24), and isoflavans (31, 34) and isoflavonoid derivatives (1, 12). The *α*-glucosidase inhibition assay was used to screen the hypoglycemic activity of the compounds. The results showed that compounds 22, 24, and 31 have a significant inhibitory effect on α-glucosidase. At a concentration of 400 μmol·L^−1^, the compound’s inhibitory effect is equivalent to that of the positive drug acarbose. An et al. (2023) isolated seven compounds from the roots of *A. membranaceus* var. *mongholicus.* anddetermined whether the monomer compounds could enhance the survival rate of HK-2 cells under high-glucose conditions as well as analyzing the effects of these compounds on the levels of the inflammatory factors tumor necrosis factor-α (TNF-α) and IL-6 using an enzyme linked immunosorbent assay (ELISA). Compounds 43–47 were found to increase the survival rate of HK-2 cells. At a concentration of 50 μmol/L, all compounds except for compound 47 significantly inhibited the elevation of TNF-α and IL-6 (p < 0.01), thereby reducing inflammatory damage in HK-2 cells. Yao et al. (2020) extracted and isolated seven flavonoids (25, 44, 46, 48–51) from the flowers of *A. membranaceus* var. *mongholicus.* They measured the ability of these compounds to scavenge DPPH and ABTS free radicals to evaluate their antioxidant capacity. The results indicated that compounds 48 and 49 exhibited strong antioxidant capacity, with IC_50_ values less than 10 μg·mL^−1^ compound 25 demonstrated an antioxidant capacity equivalent to that of the reference substance (vitamin C). In contrast, compound 50 showed poor antioxidant effects.

**TABLE 1:** Flavonoid and biological activities identified in *Astragalus* species over the past 5 years.

No.	Compound	Molecular weight	Molecular formula	Biological activity	Source and part	Ref.
1	(6a*R*,11a*R*)-3-hydroxy-9,10dimethoxypterocarpan	300	C_17_H_16_O_5_	tyrosinase inhibition, *α*-glucosidase inhibitory activity, antioxidation	B	Manh Khoa et al. (2023)
2	(6a*R*,11a*R*)-3,8-dihydroxy-9,10-dimethoxypterocarpan	316	C_17_H_16_O_6_	tyrosinase inhibition	B	Manh Khoa et al. (2023)
3	7-*β*-D-glucopyranosyloxy-astrapterocarpan	448	C_22_H_24_O_10_	—	C	Bao et al. (2022)
4	Astrapterocarpanoside A	594	C_28_H_34_O_14_	—	C	Bao et al. (2022)
5	Astrapterocarpanoside B	756	C_34_H_4_4O_19_	—	C	Bao et al. (2022)
6	Astrapterocarpanoside C	770	C_38_H_42_O_17_	*α*-glucosidase inhibitory activity, antioxidation	C	Bao et al. (2022)
7	Astrapterocarpanoside D	770	C_38_H_42_O_17_	antioxidation	C	Bao et al. (2022)
8	Astrapterocarpanoside E	932	C_44_H_52_O_22_	—	C	Bao et al. (2022)
9	Astrapterocarpanoside F	932	C_44_H_52_O_22_	—	C	Bao et al. (2022)
10	Astrapterocarpanoside G	876	C_41_H_48_O_21_	—	C	Bao et al. (2022)
11	Astrapterocarpanoside H	1,184	C_56_H_64_O_28_	antioxidation	C	Bao et al. (2022)
12	Astrapterocarpanoside I	610	C_28_H_34_O_15_	—	C	Bao et al. (2022)
13	Astrapterocarpanoside J	448	C_22_H_24_O_10_	antioxidation	C	Bao et al. (2022)
14	Licoagroside D	448	C_22_H_24_O_10_	antioxidation	C	Bao et al. (2022)
15	Astrapterocarpanoside K	580	C_27_H_32_O_14_	antioxidation	C	Bao et al. (2022)
16	Alfalone	298	C_17_H_14_O_5_	anti-tumor	A	Şahin Yağlioğlu et al. (2022)
17	Formononetin	268	C_16_H_12_O_4_	—	B	Manh Khoa et al. (2023)
18	3′*-O-*methylorobol	300	C_16_H_12_O_6_	—	B	Manh Khoa et al. (2023)
19	Prunetin	284	C_16_H_12_O_5_	—	B	Manh Khoa et al. (2023)
20	Cajanin	300	C_16_H_12_O_6_	tyrosinase inhibition	B	Manh Khoa et al. (2023)
21	Santal	300	C_16_H_12_O_6_	—	B	Manh Khoa et al. (2023)
22	5′-hydroxyl-7.2′*-O-β*-D-diglucopyranosyl-4′-methoxyl-isoflavone	624	C_28_H_32_O_16_	*α*-glucosidase inhibitory activity	C	Xiao (2024)
23	7*-O-β*-D-glucopyranosyl-calycosin	446	C_22_H_22_O_10_	*α*-glucosidase inhibitory activity	C	Xiao (2024)
24	Formononetin	268	C_16_H_12_O_4_	*α*-glucosidase inhibitory activity	C	Xiao (2024)
25	Calycosin	284	C_16_H_12_O_5_	antioxidation	E	Yao et al. (2020)
26	Pendulone	316	C_17_H_16_O_6_	—	B	Manh Khoa et al. (2023)
27	Astramembraflavane A	374	C_20_H_22_O_7_	—	B	Manh Khoa et al. (2023)
28	(−)-maackiain	284	C_16_H_12_O_5_	—	B	Manh Khoa et al. (2023)
29	Astraciceran	300	C_17_H_16_O_5_	—	B	Manh Khoa et al. (2023)
30	(3*R*)-7-hydroxy-3′,4′-dimethoxyisoflavane	288	C_17_H_20_O_4_	tyrosinase inhibition	B	Manh Khoa et al. (2023)
31	isomucronulatol	304	C_17_H_20_O_5_	*α*-glucosidase inhibitory activity	B	Manh Khoa et al. (2023)
32	(*R*)-5-(6-hydroxychroman-3-yl)-2,3-dimethoxybenzene-1,4-diol	320	C_17_H_20_O_6_	—	B	Manh Khoa et al. (2023)
33	Millepurpan	334	C_18_H_22_O_6_	—	B	Manh Khoa et al. (2023)
34	(3*R*)-7-hydroxyl-2′,5′*-O-β*-D-diglucopyranosyl-3′,4′-dimethoxyl-isoflavan	642	C_29_H_38_O_16_	*α*-glucosidase inhibitory activity	C	Xiao (2024)
35	Loureirin A	286	C_17_H_18_O_4_	—	B	Manh Khoa et al. (2023)
36	(*Z*)-2,4-dimethoxy-4-hydroxychalcone	284	C_17_H_16_O_4_	—	B	Manh Khoa et al. (2023)
37	Kaempferol 3,4′-diglucoside	611	C_27_H_30_O_16_	—	B	Manh Khoa et al. (2023)
38	Complanatuside	625	C_28_H_32_O_16_	—	B	Manh Khoa et al. (2023)
39	Quercetin 3,4′-diglucoside	627	C_27_H_30_O_17_	—	B	Manh Khoa et al. (2023)
40	Kaempferol 3*-O-*neohesperidoside	595	C_27_H_30_O_15_	—	B	Manh Khoa et al. (2023)
41	Quercetin3*-O-*[*β*-d-apiofuranosyl-(1→2)-*β*-d-glucopyranoside]	597	C_26_H_28_O_16_	—	B	Manh Khoa et al. (2023)
42	Quercetin-3*-O-*(5‴'-p-hydroxybenzoyl)-*β*-D-apiofuranosyl-(1→2)-[*α*-L-rhamnopyranosyl-(1→6)]-*β*-D-galactopyranoside	862	C_39_H_42_O_22_	—	D	Khalfallah et al. (2023)
43	Rhamnocitrin⁃3*-O-α*⁃L⁃rhamnopyronosiyl⁃(1→2) ⁃[ 6*-O-*acetyl] *β*⁃D⁃glucopyranoside	650	C_30_H_34_O_6_	anti-inflammatory	C	An et al. (2023)
44	Kaempferol⁃3*-O-β*⁃D⁃glucopyranoside	448	C_21_H_20_O_11_	anti-inflammatory	C	An et al. (2023)
45	quercetin⁃3*-O-β*⁃D⁃glucopyranoside	464	C_21_H_20_O_12_	anti-inflammatory	C	An et al. (2023)
46	Rhamnetin⁃3*-O-β*⁃D⁃glucopyranoside	478	C_22_H_22_O_11_	anti-inflammatory	C	An et al. (2023)
47	Rhamnocitrin⁃3*-O-β*⁃D⁃neohesperidoside	608	C_28_H_32_O_17_	anti-inflammatory	C	An et al. (2023)
48	Quercetin 3*-O-β*-D-galactopyranoside	464	C_21_H_20_O_12_	antioxidation	E	Yao et al. (2020)
49	7*-O-*methyl-kaempferol-3*-O-*galactosid	462	C_22_H_22_O_11_	—	E	Yao et al. (2020)
50	7*-O-*methylkampferol-3-O (6″*-O-*malonyl)-*β*-D-glucosid	594	C_30_H_26_O_13_	antioxidation	E	Yao et al. (2020)
51	Isorhamnetin-3*-O-*glucosid	478	C_22_H_22_O_12_	—	E	Yao et al. (2020)

Abbreviations: A, *A. leucothrix*; B, above-ground portion of *A. membranaceus*; C, root of *A. membranaceus* var. *mongholicus*; D, above-ground portion of *A. armatus subsp. numidicus* (Murb.); E, flower of *A. membranaceus* var. *mongholicu;* —, Needs further research.

### 3.2 Saponins and triterpenoids

**FIGURE 2:**
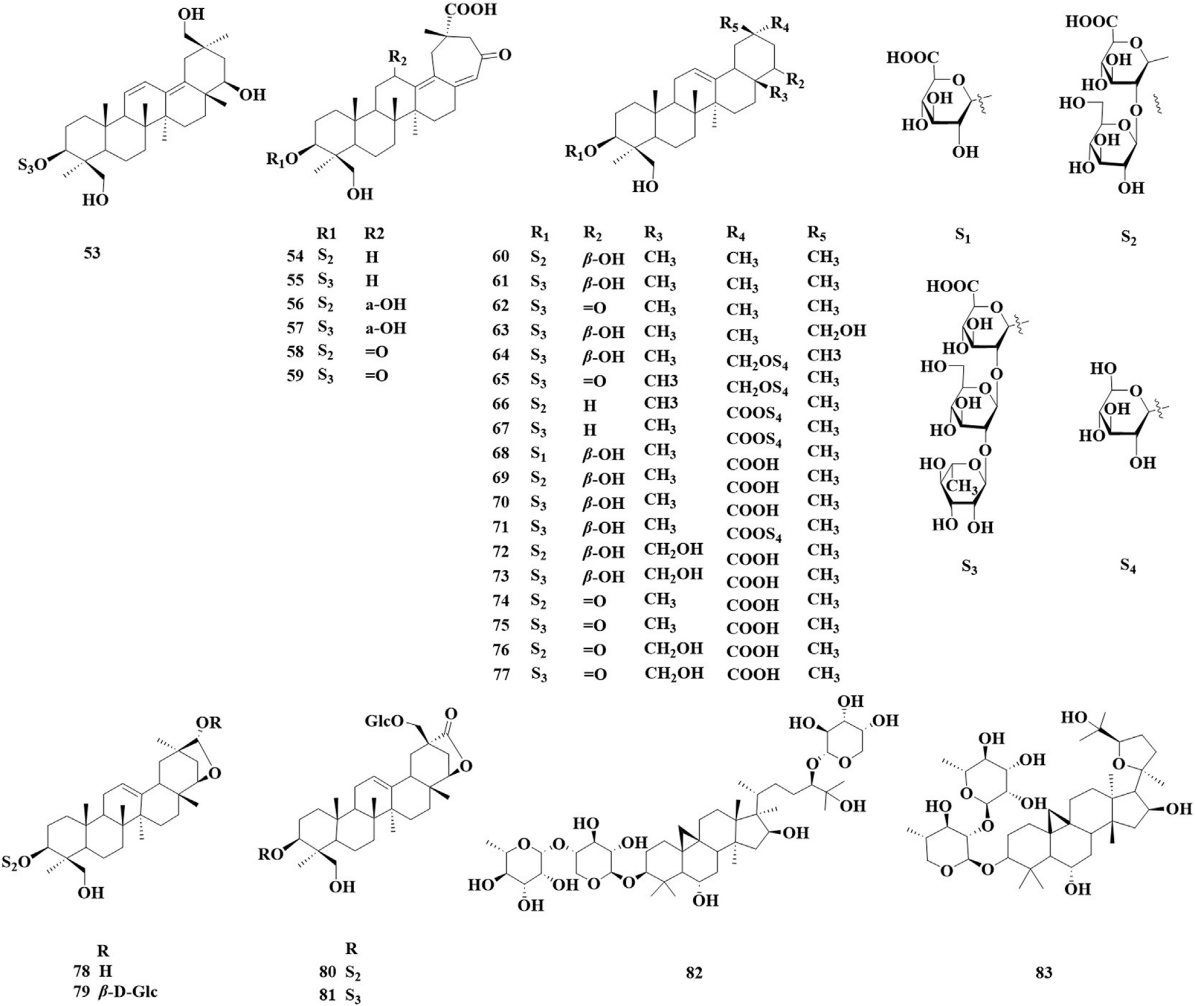
Saponin and triterpenoid compounds identified in *Astragalus* species over the past 5 years.

The number of triterpenoids isolated from the *AstragalusAstragalus* genus ranks second only to the number of flavonoids. Over the past 5 years, 31 triterpenoids have been identified in various *Astragalus* species, including oleane-type pentacyclic triterpenoids (52–80) and cycloalkane triterpenoid saponin (81, 82) (Figure 2; Table 2). These compounds exhibit biological activities *in vitro*, such as neuroprotection, antioxidant properties, and β-glucuronidase inhibition. Manh Khoa et al. (2023) isolated four new beanonide sanarosides (52, 54, 57, 58) from the ground part of A. membranaceus. Nguyen et al. (2024) isolated a total of 11 new compounds from the ground part of *A. membranaceus*, which were named them Astraoleanosides E–P (64–67, 71, 76–80), and 18 known saponins. They found that the β-glucosamidase inhibitory effect of the new compound Astraoleanoside H (67) and the well-known compound cloversaponin III (73) was comparable to that of the positive control drugs. The IC_50_ values were 21.20 ± 0.75 μM for compound 67 and 9.05 ± 0.47 μM for compound 73, with both compounds acting as competitive and non-competitive inhibitors of β-glycosalinase. Ivan Stambolov isolated a new cycloral trioxide (81) and a known cyclopenidin (82) from its overground parts of *Astragalus glycyphyllos*. The -OHDA-induced ionized brain tunnel neurotoxic, t-BuOOH-induced brain mitochondrial oxidative stress, and lipid peroxidation (non-enzyme induction) models demonstrate significant *in vitro* neuroprotective and antioxidant effects. However, when compared with the positive drug selegiline as a control, these two saponins exhibited a weaker ability to inhibit HMAO-B activity.

**TABLE 2:** Saponins, triterpenoids and biological activities of *Astragalus* species over the past 5 years.

No.	Compound	Molecular weight	Molecular formula	Biological activity	Source and part	Ref.
52	Astraoleanoside A	956	C_48_H_76_O_19_	—	A	Manh Khoa et al. (2023)
53	Astraisoolesaponin B	822	C_42_H_62_O_16_	—	A	Manh Khoa et al. (2023)
54	Astraoleanoside B	968	C_48_H_72_O_20_	—	A	Manh Khoa et al. (2023)
55	Astraisoolesaponin A2	837	C_42_H_61_O_17_	—	A	Manh Khoa et al. (2023)
56	Astraisoolesaponin A1	983	C_48_H_71_O_21_	—	A	Manh Khoa et al. (2023)
57	Astraoleanoside C	836	C_42_H_60_O_17_	—	A	Manh Khoa et al. (2023)
58	Astraoleanoside D	982	C_48_H_70_O_21_	—	A	Manh Khoa et al. (2023)
59	Azukisaponin II	797	C_42_H_68_O_14_	—	A	Manh Khoa et al. (2023)
60	Azukisaponin V	943	C_48_H_78_O_18_	—	A	Manh Khoa et al. (2023)
61	Astroolesaponins A	941	C_48_H_76_O_18_	—	A	Manh Khoa et al. (2023)
62	Astroolesaponins B	959	C_48_H_78_O_19_	—	A	Manh Khoa et al. (2023)
63	I	1,121	C_54_H_88_O_24_	—	A	Nguyen et al. (2024)
64	Astraoleanosides E	1,119	C_54_H_86_O_24_	—	A	Nguyen et al. (2024)
65	Astraoleanosides F	972	C_48_H_76_O_20_	—	A	Nguyen et al. (2024)
66	Astraoleanosides G	1,119	C_54_H_86_O_20_	—	A	Nguyen et al. (2024)
67	Astraoleanosides H	664	C_36_H_56_O_11_	inhibit the activity of *β*-glucuronidase	A	Nguyen et al. (2024)
68	II	826	C_42_H_66_O_16_	—	A	Nguyen et al. (2024)
69	Robinioside B	973	C_48_H_76_O_20_	—	A	Nguyen et al. (2024)
70	III	1,135	C_54_H_86_O_21_	—	A	Nguyen et al. (2024)
71	Astraoleanosides I	842	C_42_H_66_O_17_	—	A	Nguyen et al. (2024)
72	Astraoleanosides K	988	C_48_H_76_O_21_	—	A	Nguyen et al. (2024)
73	Cloversaponin III	825	C_42_H_64_O_20_	*inhibit the activity of β-glucuronidase*	A	Nguyen et al. (2024)
74	IV	971	C_48_H_74_O_20_	—	A	Nguyen et al. (2024)
75	Astroolesaponins E1	841	C_42_H_64_O_21_	—	A	Nguyen et al. (2024)
76	Astraoleanosides L	986	C_48_H_74_O_21_	—	A	Nguyen et al. (2024)
77	Astraoleanosides M	956	C_48_H_76_O_19_	—	A	Nguyen et al. (2024)
78	Astraoleanosides N	1,119	C_54_H_86_O_20_	—	A	Nguyen et al. (2024)
79	Astraoleanosides O	986	C_48_H_74_O_21_	—	A	Nguyen et al. (2024)
80	Astraoleanosides P	1,133	C_54_H_84_O_25_	—	A	Nguyen et al. (2024)
81	V	903	C_46_H_78_O_17_	Antioxidation and neuroprotective activity	B	Stambolov et al., 2023
82	Astrachrysoside A	769	C_41_H_68_O_13_	Antioxidation and neuroprotective activity	B	Stambolov et al., 2023

Abbreviations: Above-ground portion of *A. membranaceus*; B, above-ground portion of *A. glycyphyllos*; I, 3*-O-*[*α-*L-rhamnopyranosyl-(1→2)-*β*-D-glucopyranosyl-(1→2)-*β*-D-glucuronopyranosyl]-29*-O-β*-D-glucopyranosyl-3*β*,22*β*,24,29-tetrahydroxyolean-12-ene; II, 3*-O-*[*β*-D-glucopyranosyl-(1→2)-*β*-D-Glucuronopyranosyl]-3*β*,22*β*,24trihydroxyolean-12-en-29-oicacid; III, 3*-O-*[*α-*L-rhamnopyranosyl-(1→2)-*β*-D-glucopyranosyl-(1→2)-*β*-D-glucuronopyranosyl]-29*-O-β*-D-glucopyranosyl-3*β*,22*β*,24-trihydroxyolean12-en-29-oicacid; IV, 3*-O-*[*α*-L-rhamnopyranosyl-(1→2)-*β*-D-Glucopyranosyl-(1→2)-*β*-D-Glucuronopyranosyl]-3*β*,24-dihydroxyolean-12-en-22-oxo-29-oicacid; V, 3*-O-*[*α*-L-rhamnopyranosyl-(1→2)]-*β*-D-xylopyranosyl]-24*-O-α*-L-arabinopyranosyl-3*β*,6*α*,*β*,24(*R*),25-pentahydroxy-20*R*-cycloartane; —, Needs further research.

### 3.3 Alkaloids and simple phenolic components

**FIGURE 3:**
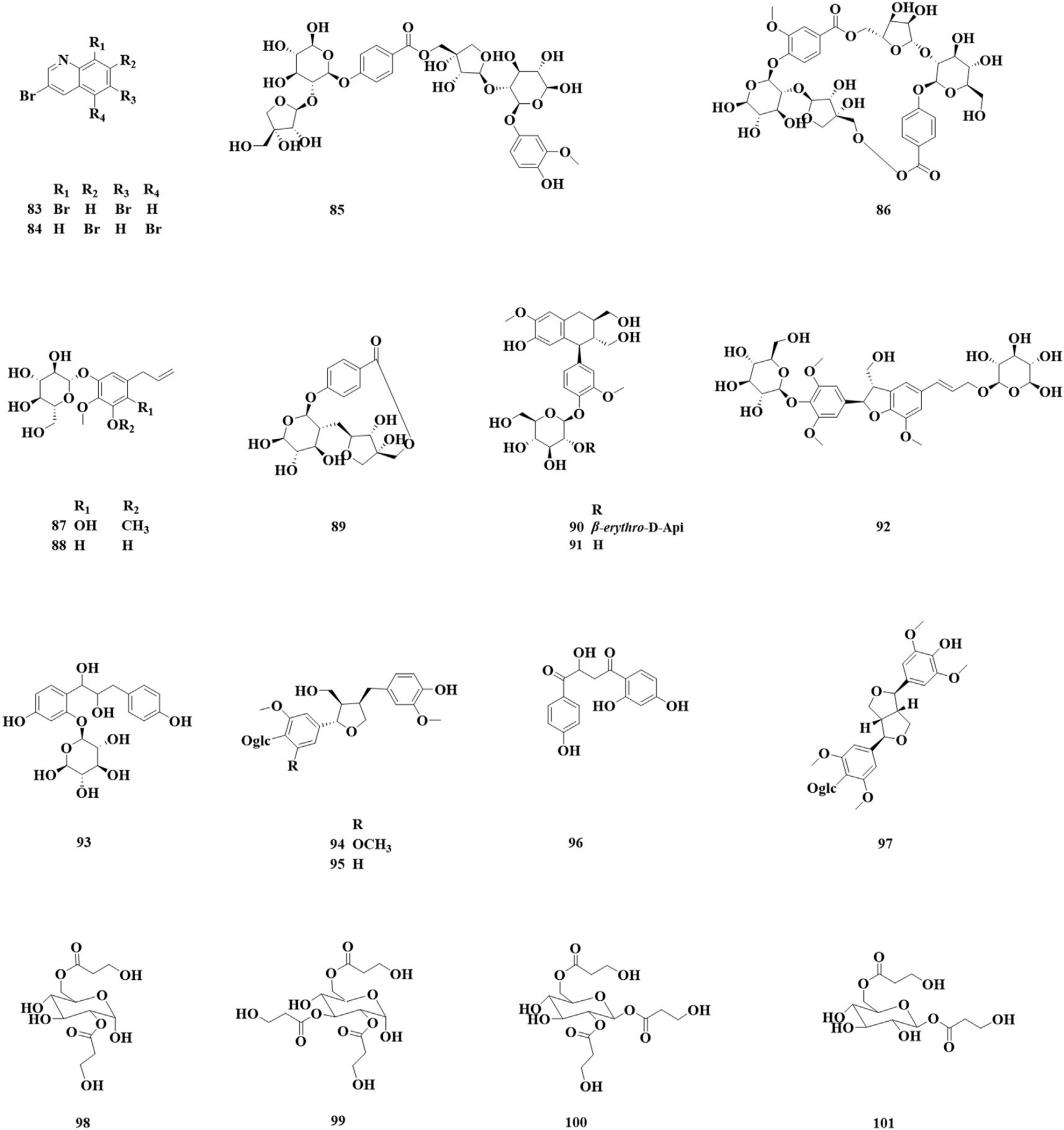
Saponin and triterpenoid compounds identified in *Astragalus* species over the past 5 years.

Nineteen alkaloids (83, 84) and simple phenolic components (85–100) have been identified in *Astragalus* species (Figure 3; Table 3). They exhibit various biological activities, including anti-cancer, peripheral analgesic, antioxidant, anti-tumor, and α-glucosidase inhibitory activities, as well as cytotoxic effects. Şahin yağlioğlu (Şahin Yağlioğlu et al., 2022) reported the separation of a new alkaline compound and a known alkaloid compound from the above-ground part of *Astragalus leucothrix*. The mixture exhibited anti-cancer activity against C6 cells (83, 84). The cytotoxic activity test indicated that a mixture of compounds 83 and 84 demonstrated significant anti-cancer activity, with an IC_50_ of 4.33 ± 0.00 μM, which is lower than that fluorouracil (5-FU). Xiao (2024) obtained roots of *A. membranaceus* var. *mongholicus*, and isolated four new phenylpropanoid compounds (85–87, 93) and five known phenylpropanoid compounds (88–92), including compound 92 from the roots, which was isolated for the first time. An *α*-glucosidase inhibition assay was conducted to evaluate the hypoglycemic activity of these compounds. The results indicated that all compounds exhibited varying degrees of α-glucosidase inhibitory activity, with the inhibition ratio ranging from 20% to 40%. Chen isolated three lupin compounds (94, 95, 97) from the roots of stem-free *A. acqualis* to conduct twisting experiments, hot water tailing tests on mice, and evaluations of 1,1-diphenyl-2-picrylhydrazyl (DPPH) free radical scavenging activity. The results demonstrated that compound 94 significantly inhibited the twisting reactions of mice during acetic acid-induced writhing tests, achieving a suppression rate of 78.8%. Additionally, in the tailing experiment, compound 97 notably increased the pain threshold of the mice, indicating significant peripheral analgesic activity. In a DPPH free radical clearance assay, compound 95 and its derivatives exhibited substantial antioxidant activity, with average IC_50_ values of 38.40 and 36.53 μg/mL, respectively. Qiu et al. (2021) isolated four hydroxy propionylated glucose derivatives from the roots of *Astragalus bhotanensis*, which were named astrabhotins A–D (98–101). The analgesic activity of compound 98 was evaluated using an acetic acid-induced peristalsis test, revealing that its analgesic effect surpassed that of the positive control, aspirin, with an inhibition rate of 52.5%. Furthermore, compounds 98 and 100 exhibited significant antioxidant activity (IC_50_ = 7.9–9.9 μM) while demonstrating weak to moderate cytotoxicity towards HepG2 cells (IC_50_ = 42.0–106.6 μM). Xiao Jun isolated a novel diphenylbutanedione (96) from the roots of A. mongholicus and employed the methylthiazolyldiphenyl-tetrazolium bromide (MTT) assay to evaluate the effects of compound 96 on the human cancer cell lines A549 (a type of non - small cell lung cancer cells), BEL-7402 (a type of human liver cancer cells), and SGC-7901 (a type of human gastric cancer cells). The results indicated that compound 96 exhibited strong cytotoxic activity against A549 cells, with an IC_50_ value of 11.41 μM. Furthermore, compound 96 inhibited the growth of BEL-7402 and SGC-7901 cells, with IC_50_ values of 36.28 μmol·L^−1^ and 45.37 μmol·L^−1^, respectively.

**TABLE 3:** Alkaloids, simple phenolic and biological activities of *Astragalus* species. over the past 5 years.

No.	Compound	Molecular weight	Molecular formula	Biological activity	Source and part	Ref.
83	3,6,8-tribromoquinoline	363	C_9_H_4_Br_3_N	anticancer activity	A	Şahin Yağlioğlu et al. (2022)
84	3,6,8-tribromochromenium	363	C_9_H_4_Br_3_N	anticancer activity	A	Şahin Yağlioğlu et al. (2022)
85	I	848	C_36_H_48_O_23_	*α*-glucosidase inhibitory activity	B	Xiao (2024)
86	II	858	C_37_H_46_O_23_	*α*-glucosidase inhibitory activity	B	Xiao (2024)
87	(2*S*,3*R*,4*S*,5*S*,6*R*)-2-(5-allyl-4-hydroxy-2,3-dimethoxyphenoxy)-6-(hydroxymethyl) tetrahydro-2H-pyran-3,4,5-triol	372	C_17_H_24_O_9_	*α*-glucosidase inhibitory activity	B	Xiao (2024)
88	Shalleroside A	342	C_16_H_22_O_8_	*α*-glucosidase inhibitory activity	B	Xiao (2024)
89	Astramemoside A	414	C_18_H_22_O_11_	*α*-glucosidase inhibitory activity	B	Xiao (2024)
90	(+)-(7*S*,8*R*,8*R*)-isolariciresinol-4*-O-β*-*D*-apiofuranosyl (1→2)-*β-D*-glucopyranoside	654	C_31_H_42_O_15_	*α*-glucosidase inhibitory activity	B	Xiao (2024)
91	Isolariciresinol 4*-O-β*-*D*-glucopyranoside	522	C_26_H_34_O_11_	*α*-glucosidase inhibitory activity	B	Xiao (2024)
92	Viburfordoside A	712	C_33_H_44_O_17_	*α*-glucosidase inhibitory activity	B	Xiao (2024)
93	(2*S*,3*R*,4*S*,5*S*,6*R*)-2-(2-(1,2-dihydroxy-3-(4-hydroxyphenyl)propyl)-5-hydroxyphenoxy)-6-(hydroxymethyl)tetrahydro-2H-pyran-3,4,5-triol	438	C_21_H_26_O_10_	*α*-glucosidase inhibitory activity	B	Xiao (2024)
94	5-methoxylarch-4*-O-β*-D-glucoside	553	C_27_H_36_O_12_	peripheral analgesic activity	C	Chen et al. (2022)
95	Larch alcohol-4*-O-β*-D-glucoside	523	C_26_H_34_O_11_	antioxidation	C	Chen et al. (2022)
96	1-(4-hydroxyphenyl)-4-(2,4-hydroxyphenyl)-2-hydroxy-1	302	C_16_H_14_O_6_	anti-tumor activity	E	Xiao Jun et al. (2022)
97	Syringaresinol-4*-O-β*-D-glucoside	580.58	C_28_H_36_O_13_	peripheral analgesic activity	C	Chen et al. (2022)
98	Astrabhotins A	338	C_13_H_22_O_10_	analgesic activity, antioxidation, cytotoxicity	D	Qiu et al. (2021)
99	Astrabhotins B	410	C_16_H_26_O_12_	—	D	Qiu et al. (2021)
100	Astrabhotins C	396	C_15_H_24_O_12_	antioxidation, cytotoxicity	D	Qiu et al. (2021)
101	Astrabhotins D	324	C_12_H_20_O_10_	—	D	Qiu et al. (2021)

Abbreviations: Above-ground portion of *A. leucothrix*; B, root of *A. membranaceus* var. *mongholicus*; C, root of *A. acaulis*; D, root of *A. bhotanensis*; E, root of *A. membranaceus*; I, ((3*S*,4*R*,5*S*)-5-(((2*S*,3*R*,4*S*,5*S*,6*R*)-4,5-dihydroxy-2-(4-hydroxy-3-methoxyphenoxy)-6-(hydroxymethyl)tetrahydro-2H-pyran-3-yl)oxy)-3,4-dihydroxytetrahydrofuran-3-yl)methyl 4-(((2*S*,3*R*,4*S*,5*S*,6*R*)-3-(((2*S*,3*R*,4*R*)-3,4-dihydroxy-4-(hydroxymethyl)tetrahydrofuran-2-yloxy)-4,5-dihydroxy-6)(hydroxymethyl)tetrahydro-2H-pyran-2-yl)oxy)benzoate; II, (12*S,*13*R*,14*S*,32*S*,33*R*,34*S*,35*S*,36*R*,92*R*,93*S*,94*R*,112*S*,113*R*,114*S*,115*S*,116*R*)-13,14,34,35.93,94,114,115-octahydroxy-36, 116-bis(hydroxymethyl)52-methoxy-12, 13,14,15,33,34,35,36,92,93,94,95,113,114,115,116-hexadecahydro-32H, 112H-2,4,7,10,12,15-hexaoxa-3. 11(3.2)-dipyrana-1(2.4),9(4.2)-difurana-5. 13(1.4)-dibenzenacyclohexadecaphane-6, 14-dione; —, Needs further research.

### 3.4 Polysaccharides

Knowing the structural components of polysaccharides is essential for understanding their composition, characteristics, and potential biological activity. A study by Liang (Liang et al., 2014) investigated the application of acid-hydrolyzed hydrophilic interaction liquid chromatography–mass spectrometry (HILIC–MS) method for analyzing the structural components of *Astragalus* polysaccharides. Utilizing a “bottom-up” approach, the researchers first optimized the conditions for the acid hydrolysis of polysaccharides derived from *Astragalus*. Subsequently, hydrophilic chromatography–mass spectrometry was employed to analyze the hydrolysis products. The findings indicated that *Astragalus* polysaccharides primarily consist of linear glucose units connected by 1 → 4 linkages, and the hydrolysis yielded polymers with degrees of polymerization ranging from 4 to 11. Wang et al. (2018) investigated the antioxidant activity of *Astragalus* polysaccharides with varying molecular weights. A range of analytical techniques, including nuclear magnetic resonance (NMR), fourier transform infrared spectroscopy (FT–IR), and gas chromatography mass spectrometry (GC/MS), were employed to characterize the products of *Astragalus* polysaccharides degradation *via* hydrogen peroxide treatment. Three molecular weights of the degraded *Astragalus* polysaccharides **(**APS) were identified 8.38, 4.72, and 2.60 kDa. No significant differences were observed in the main chain structures of the three APS variants, although slight changes in the composition of single sugar components were noted. Low-molecular-weight APS exhibited challenges in forming an active aggregation structure, resulting in less effective exposure of active groups due to unique key connections and three-dimensional structural constraints. Conversely, APS2, which has a moderate molecular weight, demonstrated the strongest antioxidant activity, effectively enhancing Superoxide dismutase (SOD) activity, inhibiting the release of malondialdehyde (MDA), and restoring cellular morphology.

In addition, Zhu et al. (2017) investigated the characterization and lymphocyte proliferation activity of oligosaccharides derived from *Astragalus* polysaccharides. *Astragalus* oligosaccharides (AOSs) were prepared *via* the acid hydrolysis of APSs. Their structure was characterized using monosaccharide analysis, periodic acid oxidation–Smith degradation, and NMR. The recovery of AOSs from cyclophosphamide-induced immunosuppression was examined using animal experiments. The findings indicate that the AOSs are octasaccharides composed of specific monosaccharides, which can restore immune function by stimulating granulocyte-macrophage colony-stimulating factor (GM-CSF) secretion. Another study (Long et al., 2024) investigated the chemical structure of APS-D1 using molecular weight distribution, monosaccharide composition, infrared spectrum analysis, methylation analysis, and NMR. It was found that APS-D1 has an average molecular weight of 7.36 kDa. Additionally, aquarium glycogen was utilized to promote fatty acid oxidation while inhibiting glycogenesis, leading to improvements in the weight of mice fed a high-fat diet (HFD), as well as enhanced sugar tolerance, insulin resistance, blood lipid disorders, and immune function. Furthermore, aquarium glycogen improved inflammation, liver and kidney function, and tissue injury; promote GLP-1 secretion; and regulate the expression of related proteins. This evidence suggests that aquarium glycogen can enhance fatty acid oxidation and mitigate the effects of low-dose dual treatments. The structural characteristics of *Astragalus* polysaccharides are crucial for elucidating their biological activity and therapeutic potential. Various analytical technologies and methodologies have been employed to elucidate the intricate structures of polysaccharides derived from *Astragalus* and other plants. Continued research in this area is vital to uncover the full potential of polysaccharides in diverse biomedical applications.

## 4 Medicine and food homologous (MFH) applications

In 2017, *Astragalus* was recognized as a functional food in China. In 2023, the National Health and Health Commission and the State Administration for Market Regulation officially included nine materials, such as *Astragali radix*, *Codonopsis radix*, and *Cistanches herba* (desert), in the traditional food material directory of Chinese medicinal ingredients (Division, 2023). As of June 2024, a query of the State Administration for Market Regulation (Regulation, S.a.F.M) reveals that *Astragalus* is categorized under health food options. Currently, there are 491 types of health foods, with 16 types classified as “national food and health characters” and 470 types categorized as “national food health” and “healthy food” VITALITY brand American ginseng, along with *Astragali Radix*, Lycium, and Polyshouwu capsules [Weishi Jianzi (2002) No. 0456] (Shenzhen Vigour Bio-health Technology Co, 2002), provide health benefits in the form of immunoregulatory effects. Additionally, Yumiao brand Huangqi Lugui wine [Weishi Jianzi (2003) No. 0202) (Changzhou Xinbo Longquan Wine Co, 2003] is noted for its anti-fatigue properties. Qinchi brand *Salvia miltiorrhizae* and *Astragali* liquor also contribute to immune regulation [Weishi Jianzi (2002) No. 0320] (Shandong Qinchi Wine Industry Co, 2017). Furthermore, the Qiling brand oral liquid Huangqi Ejiao is recognized for its ability to improve nutritional anemia (China health food approval number G20050350) (Zhangshu Qiling Pharmaceutical Co, 2019). Runhuitang brand Ejiao and Astragali liquor is associated with antioxidant and immunity-enhancing healthcare functions (China health food approval number G20110208) (Shandong Dong’e Xiuyuan Ejiao Biological Group Co, 2016). Tongrentang brand *Astragali Radix* and hawthorn tea (G20100794) (Beijing Tongrentang Xing’an Health Care Technology Co, 2020) is effective for purging, while Hefei brand *Astragali Radix* chromium yeast capsules (G20240119) (Shenyang Xiehe Group Co, 2024) aid in maintaining healthy blood glucose levels, enhance immunity, and improve nutritional anemia, conclusions supported by animal experimental evaluations. Lastly, Dangshen Bird’s Nest Huangqi oral liquid (National Food Health Note G20080250) (Zhuhai Zhengtai Pharmaceutical Co, 2021) has been increasingly recognized for its immune-enhancing effects. Different traditional Chinese medicine production practices result in varying effects, such as enhanced efficacy, altered medicinal properties, and reduced side effects, depending on factors such as the methods and auxiliary materials used. Given the differential biases and efficacies associated with Buyi, numerous approaches have been developed to formulate variety of products, to improve its effectiveness. The *Astragali Radix* Sheng decoction tablet effectivelyt supplements qi, moistens the lungs, and nourishes Yin. When prepared as a wine decoction, *Astragali Radix* exhibit enhanced properties for invigorating qi and activating blood circulation. Stir-fried *Astragali Radix* are beneficial for supplementing qi and aiding stomach digestion, while soil-stirred *Astragali Radix* are particularly effective in strengthening the spleen and preventing diarrhea. Recent studies have demonstrated that *Astragalus* species contain various types of flavonoids and polysaccharides, as described in Section 2, as well as volatile compounds and trace elements, which may account for the differences in efficacy observed among various *Astragalus* formulations (Table 4 and 5).

**TABLE 4:** Processing methods, efficacy, and application sources of *Astragali Radix*.

Processed product name	Processing method	Reason for processing	Therapeutic syndrome	Applicable symptoms	Source references
Raw *Astragali Radix*	Wash the *Astragali Radix* roots, cut them into slices or segments, and dry them in the sun or shade	To retain the original properties of the drug	Qi deficiency syndromes	Fatigue due to qi deficiency, loss of appetite, spontaneous sweating, night sweats, chronic diarrhea with prolapse of the anus, uterine prolapse*etc.*	*Shennong Ben Cao Jing* ((Wei Dynasty) Wu, 2016a)
Stir-fried *Astragali Radix*	Cut the *Astragali Radix* into slices or segments, place them in a pot, and stir-fry over low heat until slightly yellow*.*	To enhance the effects of strengthening the spleen and stopping diarrhea and reduce the cold nature of the drug*.*	Spleen deficiency syndromes	Diarrhea due to spleen deficiency, loss of appetite*etc.*	*Chinese Pharmacopoeia* (Committee, 2020), *Zhouhou Fang* (Editorial Committee of Level 77, D.O.T.C.M. Henan University of Traditional Chinese Medicine, 2023), *Xiao’er Weisheng Zongwei Lun Fang* (Anonymous, 1985)
Honey-fried *Astragali Radix*	Cut the *Astragali Radix* into slices or segments, mix them evenly with honey, let them stand for a while, and then stir-fry them over low heat until slightly yellow*.*	To enhance the qi-tonifying effect and moisten the lungs to relieve cough*.*	Qi deficiency cough	Cough due to qi deficiency, shortness of breath and fatigue, loose stools due to spleen deficiency*.*	*Chinese Pharmacopoeia* (Committee, 2020), *Ben Cao Tong Xuan* ((Ming Dynasty) Li, 2015), *Xiao’er Yao Zheng Zhi Jue* ((Song Dynasty) Qian, 2023)
Wine-fried *Astragali Radix*	Cut the *Astragali Radix* into slices or segments, mix them evenly with yellow rice wine, let them stand for a while, and then stir-fry them over low heat until slightly yellow*.*	To enhance the effects of promoting blood circulation and dredging collaterals, tonify qi, and promote blood circulation*.*	Qi deficiency and blood stasis syndromes	Chest pain and palpitations caused by qi deficiency and blood stasis*.*	*Ben Cao Tong Xuan* ((Ming Dynasty) Li, 2015), *Chuan Xin Shi Yong Fang* ((Song Dynasty) Wu, 2016b)
Salt-fried *Astragali Radix*	Cut the *Astragali Radix* into slices or segments, mix them evenly with salt water, let them stand for a while, and then stir-fry them over low heat until slightly yellow*.*	To guide the drug to the kidneys and strengthen the kidney-tonifying effect*.*	Kidney deficiency syndromes	Low back pain due to kidney deficiency, frequent urination, enuresis*etc.*	*Ji Yan Bei Ju Fang* ((Song Dynasty) Li, 1986), *Sheng Ji Zong Lu* (Liu and Jianyu, 2024)
Earth-fried *Astragali Radix*	Cut the *Astragali Radix* into slices or segments, place them in a pot, add an appropriate amount of stir-fried earth, and stir-fry them over low heat until slightly yellow*.*	To enhance the effects of strengthening the spleen and stopping diarrhea and reduce the cold nature of the drug*.*	Spleen deficiency syndrome	Diarrhea due to spleen deficiency, loss of appetite*etc.*	*Dan Xi Xin Fa* ((Yuan Dynasty) Zhu, 2020)
*Astragali Radix* Carbon	Cut the *Astragali Radix* into slices or segments and stir-fry them over low heat until they are charred black on the outside but still yellow on the inside*.*	To enhance the hemostatic effect*.*	Qi deficiency and bleeding syndrome	Bleeding due to qi deficiency, such as collapse, blood in the stool, blood in the urine*etc.*	*Chinese Pharmacopoeia* (Committee, 2020), *Hong Shi Ji Yan Fang* ((Song Dynasty) Wu, 2017), *Ji Yin Gang Mu* (Wu, 1995)

**TABLE 5:** Processing methods, efficacy, applications, and sources of *Astragali Complanati* Semen.

Processed product name	Processing method	Reason for processing	Therapeutic syndrome	Applicable symptoms	Source references
Raw *Astragali Complanati* Semen	Wash the *Astragali Complanati* Semen and dry it in the sun or shade	Retain the original properties of the drug	Kidney deficiency syndromes	Low back pain due to kidney deficiency, spermatorrhea, premature ejaculation, turbid urine, blurred vision, and excessive tearing	*Lin Zheng Zhi Nan Yi An* ((Qing Dynasty) Ye, 2021)
Stir-fried *Astragali Complanati* Semen	Put the *Astragali Complanati* Semen into a pot and stir-fry it over low heat until slightly yellow	Enhance the effect of strengthening the spleen and stopping diarrhea, and reduce the cold nature of the drug	Spleen deficiency syndromes	Diarrhea due to spleen deficiency, loss of appetite*etc.*	*Chinese Pharmacopoeia* (Committee, 2020), *Rui Zhu Tang Jing Yan Fang* ((Qing Dynasty) Ji, 2016)
Salt-fried *Astragali Complanati* Semen	Mix the *Astragali Complanati* Semen evenly with salt water, let it stand for a while, and then stir-fry it over low heat until slightly yellow	Guide the drug to the kidney and strengthen the kidney-tonifying effect	Kidney deficiency syndromes	Low back pain due to kidney deficiency, spermatorrhea, premature ejaculation, turbid urine, blurred vision, and excessive tearing	*Lei Zheng Zhi Cai* ((Qing Dynasty) Lin, 2021)

## 5 Biological activity and pharmacological action of *Astragalus* species

### 5.1 Anti-inflammatory effect

The key components of *Astragalus* species that have anti-inflammatory effects include astragalosides and flavonoids. Astragaloside IV inhibits the release of inflammatory cytokines, such as indinterleukin-1β (IL-1β) and tumor necrosis factor-α (Li et al., 2017), and alters inflammatory signaling pathways, such as the NF-κB and MAPK pathways, to inhibit inflammatory reactions (Adesso et al., 2018). Additionally, it possesses antioxidant activity, enabling it to scavenge free radicals and mitigate tissue damage resulting from inflammatory reactions. Both *in vitro* and *in vivo* experiments have demonstrated that astragaloside IV can reverse the expression of inflammatory factors, including interleukin-18 (IL-18) (Yamashita et al., 2015) IL-1β, as well as Toll-like receptor 4 (TLR4), p65, and other related proteins that are upregulated due to high glucose levels, demonstrating its significant anti-inflammatory properties (Leng et al., 2018). Studies on degenerated articular cartilage cells from human knee osteoarthritis have found that astragaloside IV can enhance the expression levels of key inflammation-related proteins, including NF-κB, IL-6, and TNF-α, by modulating a series of signaling pathways. This modulation significantly reduces the expression of these proteins, thereby inhibiting the inflammatory response and exerting a protective effect on articular chondrocytes. These findings also highlight the potential of astragaloside IV for use in improving inflammatory diseases such as arthritis (Tang, 2015). Its anti-inflammatory mechanism primarily involves regulating the body’s immune and inflammatory response processes through multiple pathways. This includes inhibiting the synthesis and release of inflammatory mediators, as well as reducing the activation and aggregation of inflammatory cells. Substances such as Quercetin (Hashemi et al., 2018), artemisinin, and isorhamnetin (Zaragozá et al., 2020) can interfere with inflammatory signal conduction, thereby mitigating damage to body tissues due to inflammatory responses. This can be applied to conditions such as rheumatism and sexually acquired arthritis (Jiang et al., 2021), among other inflammatory diseases. These findings provide robust support for the anti-inflammatory properties of *Astragalus* species.

Inflammatory mediators, such as TNF-α (Alam et al., 2021) and IL-1β (Kim et al., 2015), play a significant role in the inflammatory response. The active ingredients mentioned above can inhibit the release of these inflammatory mediators through various pathways, thereby mitigating the inflammatory response. Furthermore, flavonoids positively influence the release of inflammatory mediators. For example, hesperidin can inhibit the activity of related kinases within the cell and block the transmission of inflammatory signals. Subsequently, this action reduces the expression of key enzymes involved in the synthesis of inflammatory mediators, thereby decreasing the levels of inflammatory markers, such as IL-1β. *In vitro* cell experiments have shown that hesperidin diminishes both mRNA and protein levels of ICAM-1, IL-6, and IL-8 in monocytes. In addition, flavonoids can regulate the function of immune cells and inhibit their excessive activation, thereby preventing the release of inflammatory mediators. For instance, flavonoids can inhibit macrophages from secreting TNF-α after stimulation, maintaining the inflammatory response in a relatively controllable state and preventing excessive inflammation (Wang et al., 2020).

*Astragalus* polysaccharides play a significant role in inhibiting the release of inflammatory mediators. *Astragalus* polysaccharides can regulate the immune balance in the body and activate various anti-inflammatory intracellular signaling pathways, such as the PI3K/Akt pathway. This activation promotes the production of anti-inflammatory factors while concurrently inhibiting the release of inflammatory mediators (Meng et al., 2017). In animal experiments, the administration of *Astragalus* polysaccharides resulted in a significant reduction between 20% and 40% in the levels of inflammatory factors, such as TNF-α, in both the blood and inflammatory tissues., providing strong support for the efficacy of the intervention (Liu et al., 2019). Another study demonstrated that *Astragalus* polysaccharides markedly suppressed LPS-induced production of inflammatory factors [TNF-α, IL-6, and monocyte chemoattractant protein (MCP-1)] in RAW264.7 (mouse mononuclear macrophage leukemia cells) macrophages. At the mRNA level, LPS stimulation sharply increased the expression of these factors, while *Astragalus* polysaccharides treatment reduced their expression in a concentration-dependent manner. ELISA results confirmed these findings, showing inhibition of IL-6, TNF-α, and MCP-1 secretion. This indicates *Astragalus* polysaccharides act at both transcriptional and secretory levels to attenuate inflammation. Additionally, *Astragalus* polysaccharides (200 μg/mL) lowered p-IKKα/β levels and IKKα/β phosphorylation. They also blocked LPS-induced IkBα degradation, thereby inhibiting NF-κB activation (Guangming et al., 2023). Numerous experimental studies have confirmed the effectiveness of the overall extract of *Astragalus* species in suppressing the release of inflammatory mediators. In an animal model of arthritis, levels of inflammatory mediators in joint tissue and blood were assessed after a designated period of treatment with an *Astragalus* plant extract. Symptoms of inflammation, such as redness and joint pain, showed significant improvement. This indicates that the mechanism of action through which the extract reduces the inflammatory response involves inhibition of the release of inflammatory mediators (Li W. et al., 2019).

### 5.2 Antioxidant activity

From the perspective of antioxidant mechanisms, flavonoids primarily exert antioxidant effects by scavenging free radicals. The presence of phenolic hydroxyl groups in their molecular structure enables flavonoids to donate hydrogen atoms, which can react with highly oxidative free radicals that are destructive to cells. This interaction transforms free radicals into relatively stable compounds, thereby terminating the chain reactions of free radicals and mitigating damage to cells (Lu et al., 2023).

Flavonoids can also chelate metal ions to reduce oxidative stress (Cherrak et al., 2016). Transitional metal ions, such as iron ions (Fe^2+^) and copper ions (Cu^2+^), can catalyze the production of free radicals, including hydrogen peroxide, which exacerbates oxidative damage in the body. Flavonoids can form stable complexes with these metal ions, limiting their catalytic activity and thereby indirectly inhibiting the generation of free radicals, protecting cells from oxidative damage. For instance, in a cell culture experiment, the addition of an *Astragalus* cellular system to a cell system subjected to oxidative stimuli resulted in a significant reduction in the release of free radicals induced *via* metal ion catalysis. This intervention improved cell activity and maintained normal physiological function (Huang et al., 2020).

Flavonoids can also enhance the functionality of the cell’s intrinsic antioxidant defense system by regulating the signaling pathways (Hao et al., 2021) associated with antioxidant-related mechanisms (Chen et al., 2018). For instance, they improve the activity of various antioxidant enzymes (Wang et al., 2019), including superoxide dismutase, glutathione peroxidase, and peroxiredoxin. This enhancement further aids cells in resisting oxidative damage, maintaining redox balance within the cellular environment, and protecting against oxidative stress, thereby contributing to overall health. *In vitro* experiments demonstrated that *Astragalus* total flavonoids increased the levels of glutathione reduced (GSH) and the GSH/glutathione disulfide (GSSG) ratio in 1-methyl-4-phenylpyridinium (MPP^+^)-treated SH-SY5Y cells (human neuroblastoma cells) by 30.8% and 53.9%, respectively. GSH is a crucial intracellular antioxidant. By elevating GSH levels and the GSH/GSSG ratio, *Astragalus* total flavonoids enhanced cellular antioxidant capacity, reduced reactive oxygen species accumulation, alleviated oxidative stress damage, and thereby protected neurons (Zitian et al., 2024).

Oxidative stress contributes to the progression of multiple neurological disorders. Astragaloside IV exhibits neuroprotective, antioxidant, antiapoptotic, and anti-inflammatory properties. It reduces hippocampal oxidative stress and glial activation while preventing inflammatory cell infiltration in rats (Ianara Mendonça Da et al., 2019). Another study demonstrated that astragaloside IV combined with tetramethylpyrazine improved neural function scores, reduced infarct volume, and enhanced glucose metabolism recovery in a rat model of focal cerebral ischemia-reperfusion injury. The protective mechanism likely involves antioxidant effects, Caspase-3 suppression, and Bcl-2 upregulation, thereby mitigating oxidative stress and apoptosis while protecting neurons (Jiehong et al., 2012). Clinical studies confirmed the therapeutic efficacy of astragaloside IV. This clinical trial evaluated astragaloside IV (the active component of *Astragalus membranaceus*) in 68 intracerebral hemorrhage patients (36 treatment vs. 32 controls). The treatment group showed significantly greater improvements in functional independence measure scores at weeks 4 and 12, and in glasgow outcome scale at week 12. These benefits may stem from *Astragalus membranaceus*’ anti-inflammatory and antioxidant effects, which likely reduce cerebral edema (Chunchung et al., 2012).

### 5.3 Antineoplasmic activity

Cancer is a major disease threatening human health globally and have exhibited an increasing trend annually. According to authoritative data, tens of millions of new cancer cases are reported annually worldwide (Bray et al., 2024). In China, millions of new cases are diagnosed each year. Consequently, cancer has become one of the leading causes of death (Zheng et al., 2024). This disease not only inflicts significant physical suffering on patients, manifested as weight loss, fatigue, and pain, but also severely impacts their mental health, leading to emotional issues such as anxiety and depression. Additionally, cancer imposes a substantial financial burden on patients’ families due to high treatment costs and long-term care expenses. At the societal level, the distribution and utilization of medical resources present formidable challenges (Mao et al., 2022).

Traditional tumor treatment methods primarily include surgery, radiotherapy, and chemotherapy (Miller et al., 2022). While surgery can directly remove tumor tissues, it is often challenging to completely eliminate small lesions or metastatic cancer cells, resulting in a risk of recurrence (Song et al., 2019). Radiotherapy is the use of radiation to kill cancer cells; however, the process inevitably damages the surrounding normal tissue, leading to adverse reactions such as radiation-induced inflammation and tissue fibrosis (Bocedi et al., 2019). Chemotherapy is the use of chemical agents to inhibit the growth of cancer cells, but it is associated with significant side effects, including decreased quality of life. Furthermore, the long-term use of chemotherapy can lead to the development of resistance in cancer cells, thereby diminishing treatment efficacy (Liao et al., 2018).

*Astragalus* species, which are widely utilized in traditional Chinese medicine, demonstrate unique therapeutic effects and significant therapeutic potential against tumors. *Shennongbencaojing* identifies *Astragalus* species as a primary remedy, describing their effectiveness in treating gangrene, chronic sores, pus and pain, severe diseases, five types of hemorrhoids, scrofula of the neck and axilla, supplemental deficiencies, and a range of ailments affecting children. The *Compendium of Materia Medica* states that “*Astragalus* is sweet, warms, and purifies yang. Its uses are fivefold: the first is to make up for deficiencies; the second is to replenish vitality; the third is to strengthen the spleen and stomach; the fourth is to remove muscle heat; and the fifth is to expel pus and relieve pain.” This text further elaborates on the various pharmacological effects of *A. membranaceus* ((Ming Dynasty) Li, 2014).

In the context of anti-tumor activity, *Astragalus* polysaccharides demonstrate multiple mechanisms of action. Primarily, they strongly activate the immune system, markedly increasing the activity of immune cells, including macrophages (Li Y. et al., 2019),T lymphocytes, and B lymphocytes. This bolsters the body’s anti-tumor immune surveillance and cytotoxic capabilities (Li et al., 2020). Liver cancer studies have demonstrated that administration of *Astragalus* polysaccharides in a liver cancer mouse model significantly enhanced macrophage cell phagocytosis. Additionally, there was a marked increase in the levels of cytokines, such as interleukin-12 (IL-12). These cytokines have the potential to directly or indirectly induce the death of liver cancer cells and inhibit both tumor growth and metastasis (Lai et al., 2017). Secondly, *Astragalus* polysaccharides have been shown to effectively inhibit the proliferation of tumor cells (Tao et al., 2022). They can enhance the quality of life of mice, protect the lung tissue structure, reduce inflammatory infiltration, and delay tumor metastasis to the lungs by disrupting the pre-tumor metastatic microenvironment. The underlying mechanism may involve the inhibition of myeloid-derived suppressor cell (MDSC) recruitment to the pre-pulmonary metastatic microenvironment, as well as interference with the S1PR1/STAT3 signaling pathway, thereby exerting therapeutic effects (Shen et al., 2023). Third, the induction of tumor cell apoptosis is a crucial mechanism through which *Astragalus* polysaccharides exert their anti-cancer effects. In studies involving gastric cancer cells, *Astragalus* polysaccharides were shown to activate endogenous apoptosis pathways. Following treatment with *Astragalus* polysaccharides, the cell cycle of MGC-803 cells was arrested in the S phase. Concurrently, *Astragalus* polysaccharides decrease the mitochondrial membrane potential of MGC-803 cells. As the mitochondrial membrane potential declines, the permeability of the mitochondrial membranes increases, leading to the release of cytochrome C from the mitochondria into the cytoplasm. In the cytoplasm, cytochrome C binds with the apoptosis protease activation factor-1 (APAF-1) to form an apoptosome, which further activates caspase-9 and initiates the caspase cascade, ensuring the orderly progression of apoptosis. This ultimately results in the apoptosis of MGC-803 cells (Yu et al., 2019).

Astragaloside is a well-known *Astragalus* saponins. Numerous *in vitro* and *in vivo*, experimental studies have demonstrated that itexhibits a significant inhibitory effect on the proliferation of various tumor cells (Jiang et al., 2017). Vav3 has been identified as a guanine nucleotide exchange factor that plays a crucial role in breast cancer cells. Astragaloside IV can downregulate the expression of Vav3 in MDA-MB-231 cells in a dose-dependent manner, thereby inhibiting the activation of Rac1. Concurrently, it exhibits a significant regulatory effect on ERK1/2 and JNK within the MAPK signaling pathway, effectively downregulating phosphorylated ERK1/2 and phosphorylated JNK levels in a dose-dependent manner. However, it does not significantly affect phosphorylated p38. By reducing the expression of matrix metalloproteinases (MMPs), astragaloside IV limits the capacity of cancer cells to invade and migrate into surrounding tissues, thereby further impeding tumor progression (Jiang et al., 2017). Astragaloside IV significantly inhibit the viability of gastric cancer cell lines such as BGC-823 and MKN-74 in a concentration-dependent manner while exhibiting no effect on normal gastric epithelial cells (GES-1). Additionally, astragaloside IV can reverse the induction of TGF-β1 expression, restore the expression of E-cadherin, inhibit the expression of N-cadherin and Vimentin, and prevent the transformation of gastric cancer cells into mesenchymal cells. Furthermore, triastragaloside IV can partially reverse the effects of TGF-β1 and reduce its expression. These findings suggest that astragaloside IV may inhibit gene transcription and protein synthesis related to metastasis by interfering with the signal transduction network in cancer cells, thereby limiting their invasion and metastasis capabilities. Moreover, tetraastragaloside IV can inhibit the activation of the PI3K/Akt/NF-κB signaling pathway induced by TGF-β1 and reduce the ratios of p-Akt/Akt and p-p65/p65, effectively blocking a series of downstream signaling pathways associated with the malignant phenotype of tumors. This ultimately results in the inhibition of proliferation, invasion, and migration of gastric cancer cells (Zhu and Wen, 2018).

*Astragalus* also plays a role in modulating tumor blood vessels. The growth and metastasis of tumors are closely linked to the supply of nutrients and the metastatic potential facilitated by newly formed blood vessels. *Astragalus* can inhibit the expression and secretion of related factors, such as vascular endothelial growth factor (VEGF), which are crucial for the formation of the vascular lumen. This hinders the development of tumor blood vessels, effectively cutting off the “lifeline” of the tumor and limiting its growth and metastasis (Zhang et al., 2017).

Flavonoids and related compounds promote the proliferation and enhancement of the cytotoxicity of natural killer (NK) cells’ against K562 and SMMC-7721 cells. They also regulate the expression of cell surface markers, improving the identification and killing capabilities of NK cells. Additionally, flavonoids increase the expression of the receptors CD314 and CD336, thereby enhancing the immune activity of NK cells. Furthermore, they elevate the production of IFN-γ following the stimulation of NK-92 cells by K562 cells, with the increased secretion of IFN-γ contributing to the enhancement of immune regulation and the anti-tumor functions of natural killer cells (Han et al., 2015).

### 5.4 Enhance immune regulation

As the primary line of defense against external pathogens, the immune system plays a crucial role in maintaining the overall health of organisms (Kaur and Secord, 2019). When the immune system operates normally, the human body can effectively resist diseases and maintain optimal physical health. However, when immune function becomes abnormal—either through deficiency or hyperactivity—it can lead to a range of health issues (Rankin and Artis, 2018).

*Astragalus* polysaccharides, a key active ingredient of *Astragalus*, exert immunomodulatory effects by activating multiple signaling pathways, including the NF-κB and TLR pathways. This initiates the immune cell activation, rapidly placing them in a “fight state.” In macrophages, *Astragalus* polysaccharides bind to TLR4, activating the NF-κB signaling pathway *via* either the MyD88-dependent or -independent pathway. This interaction prompts NF-κB to translocate from the cytoplasm to the nucleus, where it binds to the κB site in the promoter region of specific genes, thereby upregulating the transcription of a series of immune-related genes. These include pro-inflammatory cytokines such as TNF-α, IL-6, IL-1β, and inducible nitric oxide synthase. The substantial expression of these molecules not only enhances the phagocytic killing ability of macrophages but also recruits additional immune cells to participate in the immune response, thereby establishing a robust immune defense and effectively resisting pathogen invasion. Concurrently, the activated TLR signaling pathway promotes the maturation and activation of dendritic cells, enhances their antigen presentation capability, activates naive T lymphocytes, and initiates adaptive immune responses, forming a solid foundation for the body’s specific immune defense response (Feng et al. 2020).

Flavonoids possess distinct immune regulatory properties, particularly in their ability to suppress inflammation-related signaling pathways and mitigate oxidative stress-induced damage (Li et al., 2018). Flavonoids can enhance the phagocytic index of macrophages, as demonstrated in related animal experiments (Guo et al., 2016). A specific dose of total flavonoids from *A*. *membranaceus* (TFA) can significantly enhance the phagocytic function of mouse macrophages. This may result from flavonoids activating relevant signaling pathways within the macrophages, which boosts their phagocytic activity and enables more effective removal of foreign substances, including microorganisms and malignant cells, thereby strengthening the body’s non-specific immune defense mechanisms. Additionally, flavonoids can regulate the secretion of cytokines and mediators by macrophages. In unstimulated macrophages, TFA induces the secretion of cytokines such as TNF-α, IL-1β, IL-6, and IFN-γ. These cytokines play a crucial role in immune regulation and can promote the proliferation of T and B cells, thereby enhancing macrophage phagocytosis and the elimination of microorganisms, which positively regulates innate immune function. In lipopolysaccharides (LPS)-stimulated macrophages, TFA can dose-dependently inhibit the excessive production of these cytokines, preventing inflammatory responses that may damage normal cells and maintaining immune balance. Flavonoids also exert a regulatory effect on nitric oxide (NO) produced by macrophages, inducing the sufficient production of NO in unstimulated macrophages. As a signaling molecule, NO participates in communication between and the regulation of immune cells. In macrophages stimulated by lipopolysaccharides LPS, flavonoids can inhibit the excessive release of NO, thereby preventing potential host cell death and inflammatory tissue damage. This further underscores the dual role of flavonoids in immune regulation and inflammation control.

### 5.5 Treating Alzheimer’s disease

Alzheimer’s disease is a debilitating neurodegenerative condition that impacts millions of individuals globally (Saido, 2024). Cellular changes in the brains of individuals with Alzheimer’s disease manifest long before the onset of clinical symptoms, underscoring the significance of early intervention and prevention strategies. Recent studies indicate that targeting astrocytes may offer promising avenues for prevention and intervention of Alzheimer’s disease; however, their role remains underexplored (Verkhratsky et al., 2019).

As Alzheimer’s disease progresses, a series of significant pathological changes occur within the brain, leading to a decline in cognitive function. The first of these changes is the deposition of β-amyloid protein. Excess β-amyloid proteins gradually aggregate to form oligomers and fibrillar plaques of various shapes, which are widely distributed in critical brain regions such as the cerebral cortex and hippocampus. This accumulation not only directly compromises the integrity of neuronal cell membranes, thereby affecting the exchange of materials and signal transmission both inside and outside the cell, but it also activates microglia and astrocytes. This activation triggers a robust neuroinflammatory response, resulting in a “second blow” to surrounding neurons, which accelerates neuronal damage and death (Murphy, 2018). Excessive phosphorylation of tau protein leads to the detachment of tau from microtubules, resulting in the aggregation of tau within neurons. This accumulation forms neurofibrillary tangles which, over time, contribute to the buildup of metabolic waste, ultimately leading to neuronal death (Rafii, 2016). The negative feedback generated by the initial two factors leads to the formation of a “death spiral.” Glial cells in the brain, including microglia and astrocytes, become abnormally activated and release excessive amounts of inflammatory cytokines and chemokines, along with chemical factors and reactive oxygen species. The excessive increase of free radicals leads to oxidative stress, which further impairs various functions of nerve cells, such as reducing intracellular ATP production, which in turn affects cell membrane phospholipid asymmetry, synaptic remodeling, nerve cell mitochondrial transport, neurite length and intracellular calcium ion concentration, ultimately leading to nerve cell death and cognitive decline (Butterfield, 2018). The appearance of oxidative stress damages nerve cells and compromises the integrity of the blood-brain barrier, leading to an influx of peripheral immune cells into the brain, further amplifying the inflammatory response (Liu et al., 2022). Studies have found that the Mediterranean diet is rich in antioxidants and vitamins that reduce the risk of Alzheimer’s disease. Antioxidant vitamins such as vitamins C and E reduce oxidative stress in the brain, and high doses may reduce the risk of Alzheimer’s disease (José et al., 2014). However, the clinical efficacy of vitamin E remains controversial, with insignificant cognitive improvement. This may stem from suboptimal antioxidant application in trials or poor blood-brain barrier penetration (Tianfang et al., 2016). Finding new drugs to address this issue is urgently needed. *Astragalus* exhibits therapeutic effects across all three stages of Alzheimer’s disease. The extracts of *Astragalus* may mitigate the pathological changes associated with Alzheimer’s disease by inhibiting the production and aggregation of β-amyloid, thereby reducing its deposition in the brain. Furthermore, *Astragalus* polysaccharides activate the Nrf2 pathway, enhance the physiological functions of APP/PS1 mice, and improve their spatial learning and memory capabilities (Qin et al., 2020). The second benefit is the enhancement of mitochondrial function. *Astragalus* may promote mitochondrial autophagy by activating the PINK1/Parkin pathway, which reduces the levels of reactive oxygen species. This, in turn, inhibits the activation of NLRP3 inflammation, thereby decreasing injury and apoptosis in nerve cells (Zhang et al., 2022). Three *Astragalus* derived compounds can regulate the activity of immune cells. *Astragalus* polysaccharides have been shown to reduce obesity, liver fat degeneration, neuritis, and cognitive impairment in metabolic/PS1DE9 mice. Moreover, improvements in weight gain, insulin and leverin levels, insulin resistance, and triglycerides in the liver, along with reductions in hyperplasia and small glial cells near plaques have been observed. These treatments also positively influenced behavioral performance (Huang et al., 2017).

A clinical study investigated the efficacy of an *Astragalus*-containing formula (Huangqi Xuming Decoction) in treating Alzheimer’s disease (AD) patients with spleen-kidney deficiency syndrome. The randomized controlled trial involved 134 participants equally divided into an observation group (n = 67) receiving conventional rivastigmine therapy plus Huangqi Xuming Decoction (with high *Astragalus* content), and a control group (n = 67) receiving rivastigmine alone. After 12 weeks of treatment, the observation group demonstrated significantly better outcomes, with a higher total effectiveness rate (89.55% vs. 71.64%), improved MMSE scores (p < 0.05), reduced TCM syndrome and ADAS-Cog scores (p < 0.05), and favorable changes in biomarkers including increased 5-HT, NE, BDNF, and SOD levels along with decreased IL-6 and hs-CRP levels (all p < 0.05). These findings provide clinical evidence supporting *Astragalus*’ therapeutic potential in alleviating AD symptoms through multiple pathways (Yuan et al., 2024). Studies have demonstrated the potential therapeutic effects, neuroprotective characteristics, and ability to alleviate oxidative stress of *Astragalus*. Thus, it is essential to thoroughly investigate the mechanisms by which *Astragalus* may influence Alzheimer’s disease and to explore its potential as a natural treatment option for this debilitating condition. Integrating aspects of Chinese medicine, such as the *Astragalus* species, into research on therapeutic strategies for Alzheimer’s disease could offer innovative approaches to enhance therapeutic outcomes and improve patients’ quality of life.

### 5.6 Treating diabetes

The incidence of diabetes, a chronic metabolic disease that affects individuals worldwide, is increasing at an alarming rate (Zhou et al., 2024). According to the International Diabetes Federation (IDF), the number of individuals diagnosed with diabetes worldwide continues to increase. In 2021, this figure surpassed 537 million and is projected to reach 643 million by 2030. It may exceed 783 million by 2045 (Magliano and Boyko, 2021).

Diabetes significantly affects the lives of patients. The common symptoms, including excessive thirst, increased appetite, frequent urination, and weight loss, severely disrupt the daily routines of individuals. Additionally, prolonged hyperglycemia can lead to a range of serious complications, such as diabetic nephropathy, diabetic retinopathy, diabetic neuropathy, and cardiovascular disease (Zhou et al., 2024). These complications can lead to a gradual decline in the patient’s physical function and may even be life-threatening (Negre-Salvayre et al., 2009).

*Astragalus* extracts reduce blood sugar primarily by enhancing insulin resistance and safeguarding pancreatic β cells. Insulin resistance is characterized by reduced sensitivity of the body to insulin, which leads to a decrease in the effectiveness of insulin in facilitating glucose uptake and utilization, thereby resulting in elevated blood sugar levels (Carr, 2014). *Astragalus* components play a significant role in enhancing insulin resistance, primarily through components such as *Astragalus* polysaccharides. These polysaccharides are capable of regulating the insulin signaling pathway and improving insulin sensitivity. When insulin binds to its receptor, the receptor activates the tyrosine kinase on the substrate, which subsequently initiates a cascade of downstream signaling events. This process ultimately facilitates the translocation of glucose transporter 4 (GLUT4) from within the cell to the cell membrane, thereby increasing the cellular uptake of glucose (Tessneer et al., 2014). *Astragalus* polysaccharides may inhibit the activity of protein tyrosine phosphatase (PTP1B), thereby reducing the dephosphorylation of the insulin receptor substrate’s tyrosine phosphorylation and enhancing insulin signaling (Wu et al., 2005). *Astragalus* polysaccharides can enhance tyrosine phosphorylation of insulin receptor substrate-1 (IRS-1) in the skeletal muscles of diabetic rats. This enhancement is associated with an increase in the expression and translocation of GLUT4, which promotes glucose uptake and utilization, ultimately improving insulin resistance (Liu et al., 2010; Ye et al., 2014).

*Astragalus* polysaccharides can regulate the secretion of fatty cytokines and enhance insulin resistance. Desertin, a protein secreted by adipocytes, improves insulin sensitivity and exhibits anti-inflammatory properties (Sidibeh et al., 2018). In patients with diabetes, secretion levels of adiponectin are frequently diminished. *Astragalus* polysaccharides have been shown to promote adiponectin secretion and elevate its plasma levels, thereby enhancing insulin sensitivity. Additionally, *Astragalus* polysaccharides can inhibit the secretion of inflammatory factors, such as resistin, which reduces the interference of inflammatory responses in insulin signaling and further ameliorates insulin resistance.

Pancreatic β cells play a crucial role in insulin secretion within the pancreas. Damage to or a reduction in the function of these cells can lead to insufficient insulin secretion, resulting in diabetes. *Astragalus* exhibits a significant protective effect on islet β cells, helping to mitigate injury and preserve their normal function (Deng et al., 2021).

The antioxidant effect of *Astragalus* is a crucial mechanism for the protection of pancreatic β cells. In the context of diabetes, hyperglycemia results in elevated levels of oxidative stress and the generation of numerous free radicals, including superoxide anions and hydroxyl radicals. These free radicals can damage the cell membrane, mitochondria, and other structures of pancreatic β cells, ultimately leading to cellular damage and apoptosis (Mathews, 2015). *Astragalus* contains a variety of antioxidant components, including flavonoids and *Astragalus* polysaccharides, which can eliminate excess free radicals in the body, inhibit oxidative stress reactions, and mitigate free radical damage to pancreatic β cells (Sun et al., 2020). *Astragalus* extract promotes the activity of antioxidant enzymes, including superoxide dismutase (SOD) and glutathione peroxidase (GSH-PX), in the islets of diabetic mice. Consequently, the level of oxidative stress on islet β cells is diminished (Li, 2012).

*Astragalus* exhibits anti-inflammatory effects and can mitigate damage to pancreatic β cells resulting from inflammatory reactions. The inflammatory response is a significant factor in the pathogenesis of diabetes. The release of inflammatory mediators, such as TNF-*α*and IL-1β, can lead to inflammatory damage to pancreatic islet β cells and inhibited insulin secretion (Pei et al., 2016; Yang, 2017). *Astragalus* flavonoids and polysaccharides have been shown to inhibit the activation of inflammatory cells and reduce the release of inflammatory factors, thereby mitigating the damage caused by inflammatory reactions to pancreatic β cells. *Astragalus* polysaccharides can suppress the expression of LPS-induced inflammatory factors such as TNF-α and IL-1β in mouse pancreatic β cells, leading to a reduction in the rate of cell apoptosis and protecting pancreatic islet β cell function (Lu et al., 2013).

In a clinical study, the combination of *Astragalus* decoction pieces with conventional therapy showed positive effects on insulin secretion function and insulin resistance in type 2 diabetes mellitus (T2DM) patients. The study included 97 T2DM patients, divided into a control group (n = 48) and an observation group (n = 49). Before treatment, there were no significant differences in fasting blood glucose (FBG) and 2-h postprandial blood glucose (2h-PBG) between the two groups. After treatment, the observation group exhibited lower FBG (6.01 ± 1.99 mmol/L) and 2h-PBG (8.12 ± 1.92 mmol/L) compared to the control group (7.48 ± 1.50 mmol/L and 9.08 ± 1.53 mmol/L, respectively), with a statistically significant difference (P < 0.05). Before treatment, there were no differences in serum C-reactive protein (CRP), tumor necrosis factor-α (TNF-α), plasma insulin levels, or insulin resistance index (HOMA-IR) between the groups. However, after treatment, the observation group showed significantly lower levels in all these markers compared to the control group (P < 0.05) (Song, 2024).

## 6 Conclusion and perspectives

**FIGURE 4:**
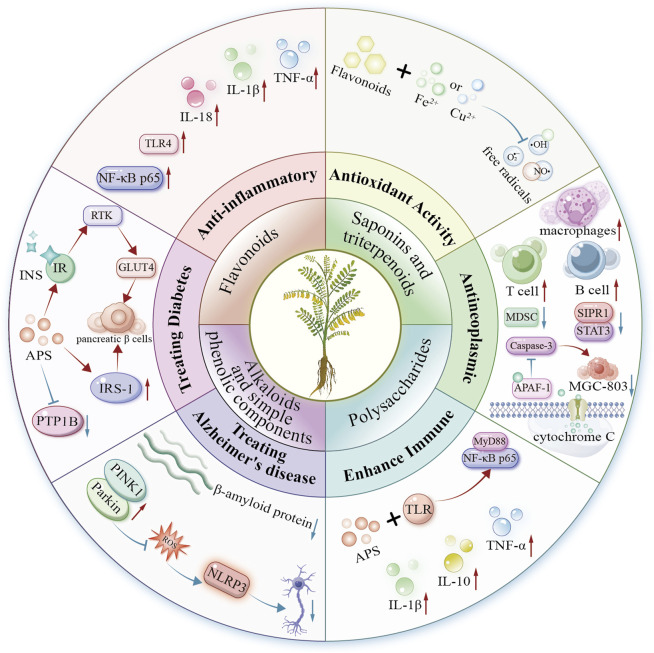
Mechanism diagram of biological activity of *Astragalus* species.

*Astragalus* L. is widely studied for its medicinal and nutritional value, especially in traditional Chinese medicine. Recent studies (2020–2025) reveal key bioactive compounds in *Astragalus* species: flavonoids, triterpenoid saponins, alkaloids, and polysaccharides. These compounds exhibit diverse pharmacological effects, including anti-inflammatory, antioxidant, immunomodulatory, antitumor, neuroprotective, and hypoglycemic activities. Their structural diversity enables multifaceted mechanisms of action. Thus, *Astragalus* is a promising resource for managing chronic diseases such as diabetes, Alzheimer’s disease, and cancer (Figure 4).

*Astragalus* species exemplifies MFH, serving as both a therapeutic agent and functional food. Processing methods like stir-frying, honey-frying, and wine-frying can enhance specific medicinal properties, broadening its applications in dietary supplements, teas, and health products. However, standardizing *Astragalus* species extracts remains challenging due to variations in plant sources, growing conditions, and extraction methods. These variations cause inconsistencies in bioactive compound composition and concentration, hindering quality control and reproducibility in research and clinical practice.

Clinical translation of *Astragalus* species faces key challenges. Current evidence mainly comes from *in vitro* and animal studies, with few high-quality human trials confirming efficacy and safety. Existing clinical data often have limitations: small sample sizes, short durations, and inadequate controls. Moreover, long-term safety of *Astragalus* species compounds—particularly at high doses or in vulnerable groups (pregnant women, children, patients with severe chronic conditions)—remains unclear. These gaps underscore the urgent need for systematic clinical safety assessments.

Future *Astragalus* research must emphasize rigorous clinical trials to confirm efficacy and establish optimal dosing. Mechanistic studies should precisely define molecular targets to develop precision therapies. Critical evaluation of drug-herb synergies is needed, particularly for cancer and metabolic diseases. Advanced omics and bioinformatics approaches will be essential to discover novel bioactive compounds and enhance existing formulations.

In summary, *Astragalus* species shows great promise as a natural therapy and functional food. However, standardization, safety assessment, and clinical validation remain key challenges for its mainstream use. Although adverse effects are usually mild, proper dosing and medical supervision are vital, especially for patients with pre-existing conditions or concurrent medications. Future work should prioritize translational research to advance lab findings into clinical and commercial applications. This approach can enhance human health while reducing risks.
